# *Humanoid* infers Archimedes' principle: understanding physical relations and object affordances through cumulative learning experiences

**DOI:** 10.1098/rsif.2016.0310

**Published:** 2016-07

**Authors:** Ajaz Ahmad Bhat, Vishwanathan Mohan, Giulio Sandini, Pietro Morasso

**Affiliations:** Robotics, Brain and Cognitive Science Department, Istituto Italiano di Tecnologia, Via Morego 30, Genova, Italy

**Keywords:** affordances, animal cognition, cause–effect relations, episodic memory, developmental robotics

## Abstract

Emerging studies indicate that several species such as corvids, apes and children solve ‘The Crow and the Pitcher’ task (from Aesop's Fables) in diverse conditions. Hidden beneath this fascinating paradigm is a fundamental question: by cumulatively interacting with different objects, how can an agent abstract the underlying cause–effect relations to predict and creatively exploit potential affordances of novel objects in the context of sought goals? Re-enacting this Aesop's Fable task on a humanoid within an open-ended ‘learning–prediction–abstraction’ loop, we address this problem and (i) present a brain-guided neural framework that emulates rapid one-shot encoding of ongoing experiences into a long-term memory and (ii) propose four task-agnostic learning rules (elimination, growth, uncertainty and status quo) that correlate predictions from remembered past experiences with the unfolding present situation to gradually abstract the underlying causal relations. Driven by the proposed architecture, the ensuing robot behaviours illustrated causal learning and anticipation similar to natural agents. Results further demonstrate that by cumulatively interacting with few objects, the predictions of the robot in case of novel objects converge close to the physical law, i.e. the Archimedes principle: this being independent of both the objects explored during learning and the order of their cumulative exploration.

‘Any floating object displaces its own weight of fluid'

—Archimedes of Syracuse (Treatise on Floating Bodies)

## 1. Introduction

Archimedes' principle is a law of physics that is fundamental to fluid mechanics. Beyond the succinct formal expression of a physical phenomenon, a more fascinating aspect is how such causal relations are approximately learnt and exploited for survival by creatures (such as animals and children) who might have never heard about such laws in the first place. A recent example comes from experiments re-enacting one of Aesop's most familiar fables that even predates Archimedes, i.e. the story of ‘The Crow and the Pitcher’. This fable has been tested on a range of corvid species particularly New Caledonian crows [1–3]. Results indicate that after exploring various objects, crows preferentially dropped large objects instead of small objects [2], sinking objects rather than floating objects and solid objects rather than hollow objects [1]: suggesting that they gradually learnt the causal relations between functional properties of objects and the resulting liquid displacement. The paradigm has been reproduced in other species—rooks [4], Eurasian jays [5] and human children [6], suggesting similar strategies used and ensuing behaviours.

Hidden underneath this playful paradigm are a set of fundamental questions that motivate this work: (i) what are the shared cognitive mechanisms in human and non-human animals which enable them to learn and infer causal relations between their *goals, actions and the objects* in their environments? (ii) By cumulatively interacting with different objects in the world, how are task-relevant physical relations and object affordances both abstracted and exploited in novel contexts? Answers to these questions will not only help shed light on the computational/neural underpinnings that not only orchestrate such cognitive behaviours in natural and artificial agents but also aid the design of intelligent robots that can learn and apply causal knowledge during diverse applications in natural living spaces [7,8]. This article is an adventurous exploration in this direction, re-enacting the open-ended loop of explorative learning–prediction–consolidation–goal-directed reasoning in the context of the Aesop's Fable paradigm on a state-of-the-art humanoid platform, iCub (see Material and methods: §5.1).

Several modelling studies have attempted to investigate and model causal cognition ranging from earlier heuristic approaches [9,10], connectionist models [11–13], and probabilistic and Bayesian networks [14–16]. In parallel, learning object–action relations and using such knowledge for prediction and planning is becoming an active topic of study in embodied robotics with modelling approaches ranging from probabilistic Bayesian networks [17–19] to support vector classifiers [20–22] and fuzzy logic systems [23–25]. While a detailed commentary on these methods is out of the scope of the present study, known bottlenecks of these methods (e.g. [26]) have been the applicability and generalization to novel contexts, new tasks especially in unstructured settings, and the ability to learn cumulatively through time and experience (like natural cognitive agents). We believe, while modelling causal learning and inference from the perspective of a cumulatively developing system (animal, embodied robot), it is critical to look at the loop of perception–action, learning, memory and goal-oriented reasoning in a holistic fashion. Further, there are relatively few studies validating these models in terms of their neural underpinnings [27], and connecting to emerging trends from neurosciences, for example, the distributed property-specific organization of sensory motor information [28,29], the discovery of the default mode network (DMN) [30,31], emerging trends from connectomics [32] and small world properties [33]. The present work closely takes inspiration from these trends to propose a novel framework for cumulative learning and abstraction of cause–effect relations. Specifically, we consider the following emerging trends from neuroscience relevant to guide the design of a neural framework that supports causal learning and inference.

### 1.1. Distributed plus hub view of semantic memory

Presently, there is mounting evidence both from functional imaging [34,35] and connectomics [33,36] suggesting that sensorimotor knowledge is grounded in a distributed fashion in property-specific cortical networks that directly support perception and action and that were active during learning [28,37]. Activations in the property-specific zones (colour, shape, word, faces, etc.) are gradually integrated [38,39] and communicate through shared multi-modal zones or hubs located in the anterior temporal lobes. Further, same network of cortical areas are active both during real perception, imagination and lexical processing with retrieval or reactivation of the neural representation triggered based on partial cues coming from multiple modalities: for example, sound of a hammer retro-activates its shape representation [40]. The core rationale of this distributed plus hub perspective is that while there is a fine level of functional segregation (property-specific processing zones) and global integration (though multi-modal hubs) in the cortical organization of semantic memory, there is also an underlying dynamics that facilitates cross-modal, top-down and bottom-up activation of these areas. The neural framework proposed in this article closely emulates this organization of conceptual memory.

### 1.2. Interplay between memory, prospection and consolidation

The discovery of the DMN [28,29] provides converging evidence that seemingly disparate cognitive functions like recalling past episodic memories, prospective simulation of the future and goal-directed planning engage a shared set of highly connected cortical areas (or hubs) in the brain. At the same time, semantic learning is known to be a fruit of abstraction and consolidation of episodic events over time [41,42]. In this sense, rapid/one-shot encoding new episodic experiences (in medial-temporal-lobe), gradual consolidation of such experiences into the semantic memory (putting the past in the context of the present) and prospection (using the present to simulate the future, generate plans) are closely linked. Thus, complementing the property-specific organization of sensorimotor information and its global integration through hubs, the proposed architecture incorporates an episodic simulation system in line with the known function of the DMN of the brain.

In brief, this article provides the following novel contributions to the existing research: (i) a neural, embodied, brain-guided, memory-based, cognitive framework for causal learning through cumulative exploration of real-world environment; (ii) the interplay between two memory systems (episodic and semantic) and how it leads to abstraction of diverse causal relations; (iii) four simple task-agnostic learning rules (elimination, growth, uncertainty, status quo) that correlate predictions emerging from past learnt experiences in the context of new/present experience to gradually abstract underlying causal relations; (iv) validation of the framework by mimicking a well-established paradigm to investigate causal learning in corvids and children; and (v) application of the model to practical robotic implementations. Importantly, the loop between learning, prediction and abstraction is always closed: more learning and experience leading to better prediction, inconsistencies in prediction leading to new learning.

The rest of the article is structured as follows. The following §1.3 gives a brief description of the Aesop's Fable paradigm, followed by §2 on the proposed computational/neural architecture and learning rules for abstraction of causal knowledge. With results from experiments on the Aesop's Fable task, §§3.1 to 3.3 progressively describe the cumulative learning and inference of diverse causal relations by the humanoid iCub through successive interactions with objects of varying physical properties. Creative exploitation of such knowledge in under novel situations and a comparison between the gradual evolution of the prediction error of the robot and the physical law is summarized in §§3.4 and 3.5. A short discussion follows.

### 1.3. Causal learning task

**Figure 1.**
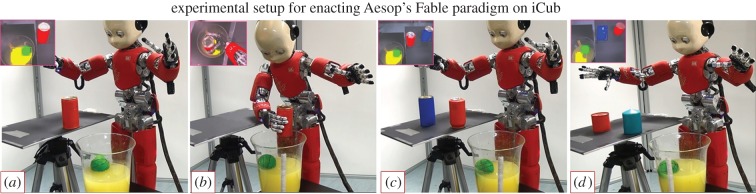
Experimental set-up for the Aesop's Fable task. The set-up consists of a jar of water (water is coloured yellow for recognition through colour perception) containing a green ball which the robot cannot reach initially. Some object(s) are available on the stand, within reach of the robot. In each experiment, the robot drops object(s) into the jar of water, raising the water level to reach the goal (green sphere). Each experiment involves either dropping a single object or making a choice between multiple objects, exhibiting causal learning and inference processes. The objects to explore vary in their physical properties: colour, size, shape and weight. The humanoid robot used in the experiments was developed at Istituto Italiano di Tecnologia (IIT) through the EU funded project RobotCub (Material and methods: §5.1).

The task is inspired by Aesop's well-known tale ‘The Crow and the Pitcher’, in which a thirsty crow drops stones into a half-filled pitcher of water, thereby raising the water level until it is high enough to drink. Recently, the paradigm has been reproduced in a range of species such as rooks [4], corvids [1], Eurasian jays [5] and human children [6] as articulated in the previous section. In an equivalent scenario (figure 1), the iCub robot is presented with a random set of objects to explore and a jar of water containing a floating target/goal (green ball). With the goal to reach the (otherwise unrealizable) green ball, iCub explores its environment to learn if the available objects can help realize its goal. We chose a real robotic platform instead of a simulation because while using a real robotic embodiment, the real world with its intricate dynamics is available for free, allowing us to conduct experiments with a range of objects with diverse physical properties on the fly in an open-ended fashion, without additional simulation infrastructure. The robot is endowed with the following basic sensorimotor capabilities to begin with (i) object perception through colour and shape (see Material and methods: §5.2–5.4); (ii) ability to perform reach–grasp actions on the objects [43], which is needed to initiate the cumulative explorative learning loop. The interested reader may refer to [43,44] for a review on the iCub action generation system. *A priori*, there is no previous experience and nothing is known about the causal nature of the task at hand.

## 2. Computational model

### 2.1. Organization of sensorimotor information through hubs

**Figure 2.**
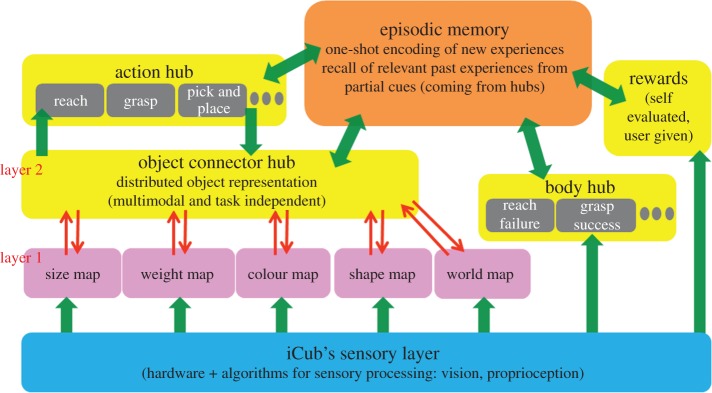
The figure shows a block diagram of how information related to perception and action are organized and their link to the robots episodic memory. There is a distributed property-specific organization of sensorimotor information. Layer 1 maps are fed bottom-up by information coming from the sensory layer and represent the properties of objects in a distributed fashion. Activations in the level 1 maps are integrated at layer 2 (i.e. object hub), leading to a multi-modal representation of the object in the scene. An episode of experience is the temporal sequence of activations in the different hubs (object, action, reward, body) when the robot engages in an interaction with the environment. Such experiences are stored in the episodic memory network through one-shot learning. Inversely, activations in the hubs also act as partial cues, to trigger retrieval of the full experience from the episodic memory (e.g. perceive a heavy cylinder and recall a past experience of dropping it in water and the ensuing consequences).

Figure 2 shows the central building blocks and information flows in the proposed framework. At the bottom is the sensory layer to analyse properties of the objects; in particular colour, shape, size and weight. Word information is an additional input coming from the teacher either to issue user goals or teach names of new objects [45]. Information coming from the sensory layer is projected bottom-up to a set of growing self-organizing maps (SOMs [39]), organized in a property-specific fashion. These maps learn and represent object properties as conceptual features. Neural connectivity between the sensory layer to the property-specific maps is learnt using standard SOM procedure (see Material and methods: 5.2–5.4). There onwards, a sensory input pertaining to a property would activate a winner neuron in the corresponding property-specific SOM. In this sense, layer 1 emulates the distributed property-specific organization of perceptual information in the brain [28,34]. For example, perceptual analysis of an object (e.g. a large heavy blue cylinder) leads to activations in different property-specific SOM's coding for colour, shape, weight and size, respectively. The local activations in the property-specific maps form the input to layer 2 SOM, i.e. the object hub. The object hub facilitates integration, leading to a final representation of the object in the scene. Note that, as we move upwards in the hierarchy, information becomes more and more integrated and multi-modal, and as we move downwards information is more and more differentiated to the level of perceived properties. The network of the object hub and property-specific maps is complemented with a network dynamics that allows the neural activity in one map to retro-activate other members of the network and hence allowing information to move top-down, bottom-up or in cross-modal fashion [45] (see Material and methods: §5.5). For example, a word like ‘green sphere’ generates activations in the word map that causes activity in the object hub, which in turn can retro-activate the colour and shape maps, similar to anticipating top-down what a ‘green sphere’ might be.

In relation to the organization of action, there is a subtle separation between the representation of actions at an abstract level (what can be done with an object) and the action planning details (how to do). While the former relates to the motor affordance of an object, the latter relates to motion planning details that is not depicted here. The interested reader may refer to [43,44] for details related to motion planning. The abstract layer corresponds to an action hub and consists of single neurons coding for different action goals such as reach, grasp, etc., and grows with time as new primitives are learnt. In this sense, neurons in this top-level action hub are similar to canonical neurons found in the premotor cortex [46] that are activated at the sight of objects to which specific actions are applicable. Finally, the consequences of both perceptions and actions alter the state of the body itself. This is represented by the body hub. In other words, the body hub is a neural map that explicitly represents the state of the body (like failing to reach an object or grasping it, or successful realization of goal). Reward of an experience is either given by the user or evaluated by the robot itself though observation. In the present task, reward is the volume/level of water raised by dropping an object into the jar of water.

### 2.2. Episodic memory system and its link to the hubs

**Figure 3.**
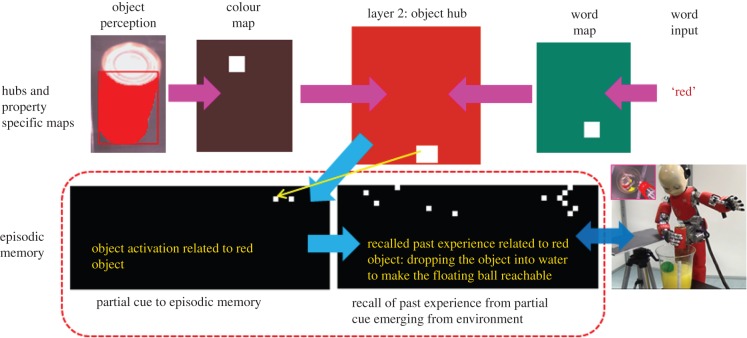
Link between episodic memory system and hubs–property-specific maps. A memory trace in the episodic memory is organized as a distributed activity in 1000 neurons arranged in a sheet-like structure with 20 rows, each containing 50 neurons. Each row corresponds to activity in either of the hubs at the time the experience was gained and the whole temporal sequence of an episode of experience. Reconstruction of a complete previously encoded episodic memory trace can be triggered by a partial cue coming from the environment (e.g. perceiving a red cylinder can generate a partial cue that recalls the past experience of dropping the red cylinder into water to make a (otherwise unreachable) floating ball reachable). The humanoid robot used in the experiments was developed at Istituto Italiano di Tecnologia (IIT) through the EU funded project RobotCub (Material and methods: §5.1).

Practically, when a humanoid robot such as iCub interacts with the environment, it is the ongoing sequences of actions on various objects, the ensuing consequences, internal body state and rewards received that mainly form the content of its experiences. Thus, in our architecture, it is the temporal sequence of activations in the different hubs (object, action, reward, body) when experience is gained that forms the content of the episodic memory. The episodic memory network to encode multiple sensorimotor experiences of iCub is realized using a recently proposed excitatory–inhibitory neural network of auto-associative memory [47,48]. For modelling purposes, the memory network consists of 1000 neurons, organized in a sheet-like structure with 20 rows each containing 50 neurons (figure 3). Every row is an event in time (related to activation in object hub, action hub, body hub or reward) and the complete structure as an episode of experience. For example, being unable to reach a floating object in a jar of water (body hub state), perceiving a red cylinder (object hub state), dropping it in water (action hub state) and fetching a reward of 15 (end reward). In the future, if the robot perceives the red cylinder, the object hub state serves as a partial cue to reconstruct the full experience. Importantly, in the memory network of 1000 neurons, multiple episodic memories can be represented and retrieved (approx. 230 episodes [48]). The learning rule to encode new experiences into the episodic memory network as well as the neural dynamics to recall an episode from partial cues is described in Material and methods: §§5.6 and 5.7.

### 2.3. Learning rules for consolidation and causal abstraction

**Figure 4.**
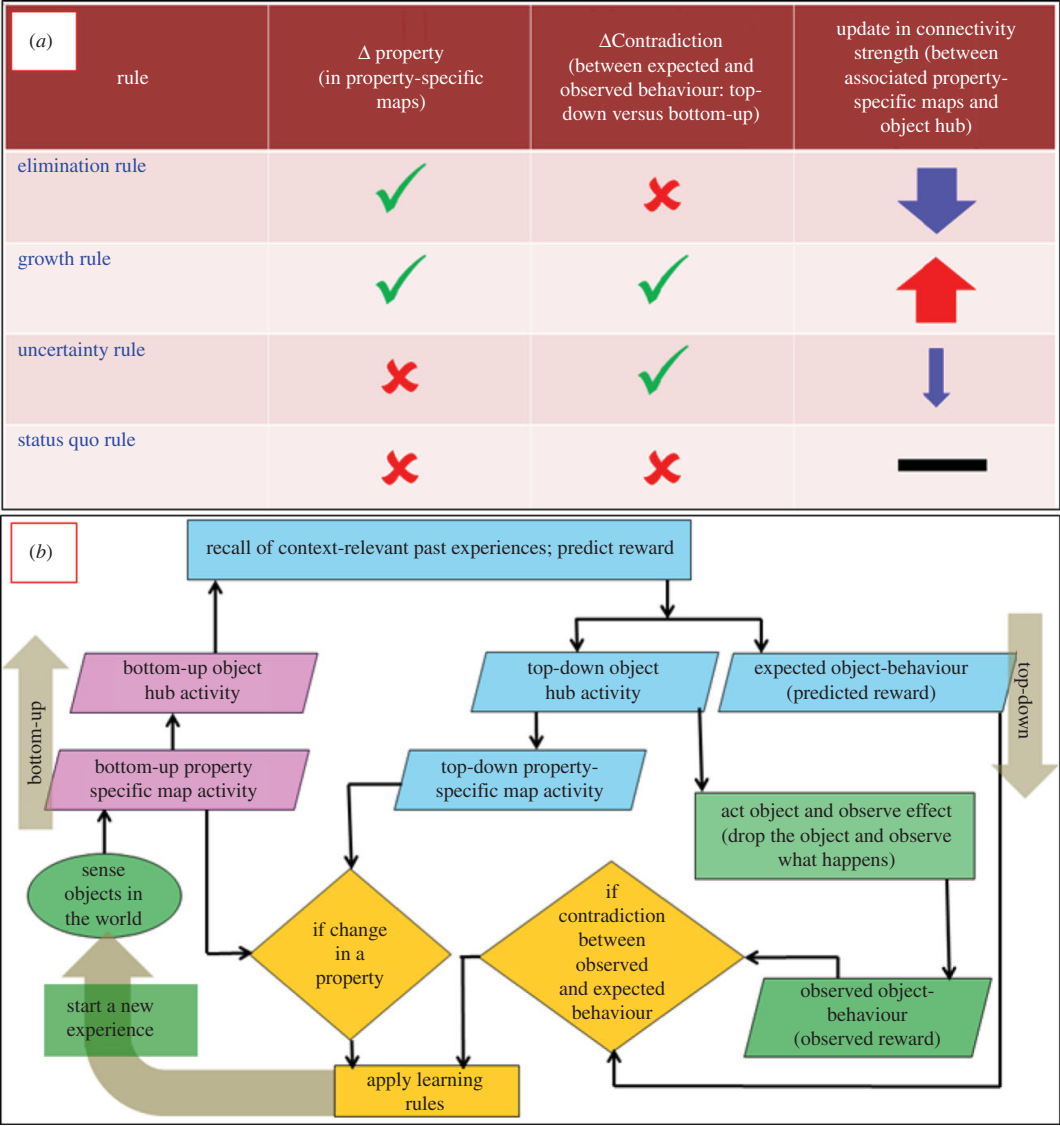
Learning rules. (*a*) Summarizes the four rules for learning cause–effect relations in a cumulative set-up. (*b*) A colour-coded flowchart of the complete loop of information processing within which the learning rules are applied. The loop connects four key processes: (1) robot's sensorimotor interaction with the world (green blocks); (2) representation and bottom-up flow of the sensory information in property-specific maps and object hub for recall of past experiences relevant to what is sensed in now (pink blocks); (3) top-down flow of recalled information from memory into the object hub, property-specific maps and reward anticipation (blue blocks) and (4) comparison of bottom-up versus top-down information at the level of property-specific maps, comparison between the anticipated reward and the reward observed through sensorimotor interaction with the world, and henceforth, application of learning rules (orange blocks). The loop begins with the robot's interaction with the world and completes with the application of learning rules (see bottom-left).

In this section, we describe four simple rules for abstraction of causal relations. Figure 4*b* gives a flow chart of how in the current architecture learning rules are applied when the robot cumulatively interacts with the world. Figure 4*a* tabulates four rules that allow us to compare what has changed between what has been experienced in the past (recalled by the episodic memory) and what is happening in the new situation. Let us consider ΔProperty as the difference in activity in a property-specific map when activated bottom-up (through sensory layer) and when activated top-down through recall of past experience by episodic memory, and ΔContradiction as the difference between the robots anticipation of how an object might behave (expected reward due to recalled past experience) and the real observed behaviour. Then the learning rules are as follows:

#### 2.3.1. Elimination rule

If 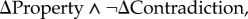 then that property is not causally dominant. The result is a drastic reduction in the connection strength between the object hub and the associated property-specific map. For example, by dropping a *red* cylinder and a *blue* cylinder and observing the underlying consequences (water displacement), the robot infers that the colour of objects is not a causally dominant property. Results section, episodes 1–2, shows several scenarios related to the application of this rule.

#### 2.3.2. Growth rule

This rule complements the elimination rule. If 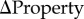 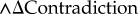, then that property is causally dominant. Contradiction in the robot's anticipation implies that there is something new that has not been learnt in the past episodes of experiences. Results section, episodes 3–4, shows scenarios related to the application of this rule.

#### 2.3.3. Uncertainly rule

If 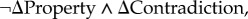 the connection strength between the object hub and the associated property-specific map is marginally reduced. This is because in this condition it is not possible to infer with certainty whether the particular property is causally dominant or not, unless further experience is gained by cumulative learning. Results section, episodes 3–4, shows scenarios related to the application of this rule.

#### 2.3.4. Status quo rule

If 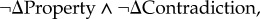 there is nothing new to learn and the world is behaving the way the robot anticipates it should, so status quo is retained (no network growth, no change in connectivity).

The next section presents results from the first episode of cumulative learning where the robot has no prior experience and the gradual encoding of new experiences into the episodic memory, the application of different learning rules and the ensuing inferences/predictions of the robot under novel situations.

## 3. Results from Aesop's Fable experiments on iCub Humanoid

Given that the robot is learning cumulatively, this section is presented in terms of episodes evolving in time. Through results emerging from every episode of gradual learning, we describe the interaction between different core elements in the computational model (property-specific maps, hubs, episodic memory) and the application of learning rules. Further, every episode has a common set of underlying computational processes, (1) bottom-up experience/interaction with the world and hence activation of various maps (see Material and methods: §§5.2–5.5); (2) recall of past experiences from memory, if any (see Material and methods: §§5.6 and 5.7); (3) use of recalled past experiences to anticipate (see Material and methods: §5.8); and (4) application of the learning rules based on the anticipation from past experience and the ensuing real observation. The underlying formal analysis of steps 1–3 is further explained in the Material and methods section. The overall sequence is depicted in figure 4*b*.

### 3.1. Learning that colour of objects is causally irrelevant to the Aesop's Fable task

#### 3.1.1. Episode 1

**Figure 5.**
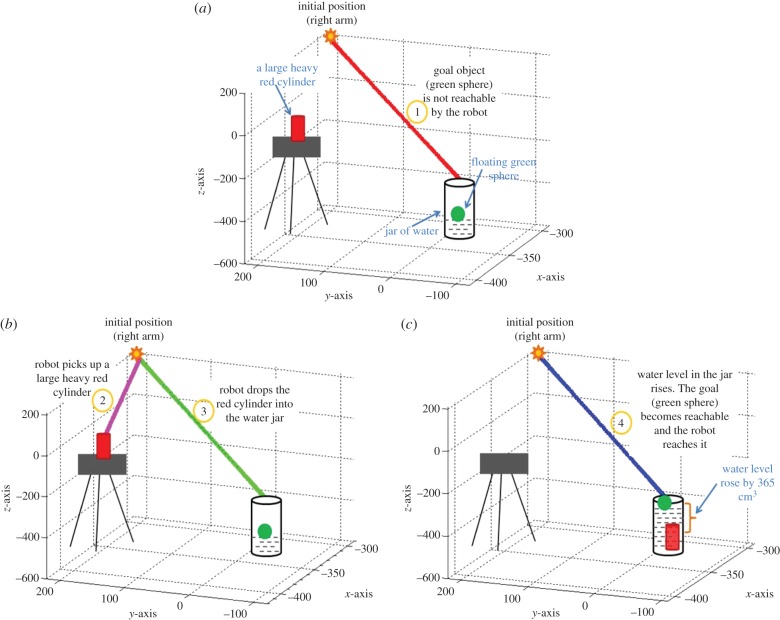
Pictorial diagram of the Aesop's Fable task. (*a*) Shows the experimental scenario where the robot is assigned the task of reaching a green sphere floating in a jar of water. The motion planning system of iCub provides the information that the goal is unreachable. (*b*) Exploring its environment, the robot finds a large heavy red cylinder and chooses randomly to pick it up and drop it into the jar. (*c*) The robot observes that dropping the object into jar displaced the water by a volume of about 365 cm^3^. Returning to its original goal, the robot finds the green sphere is now reachable, reaches the goal and encodes this experience of ‘making an unreachable goal reachable’ into episodic memory for recall and re-use in future.

**Figure 6.**
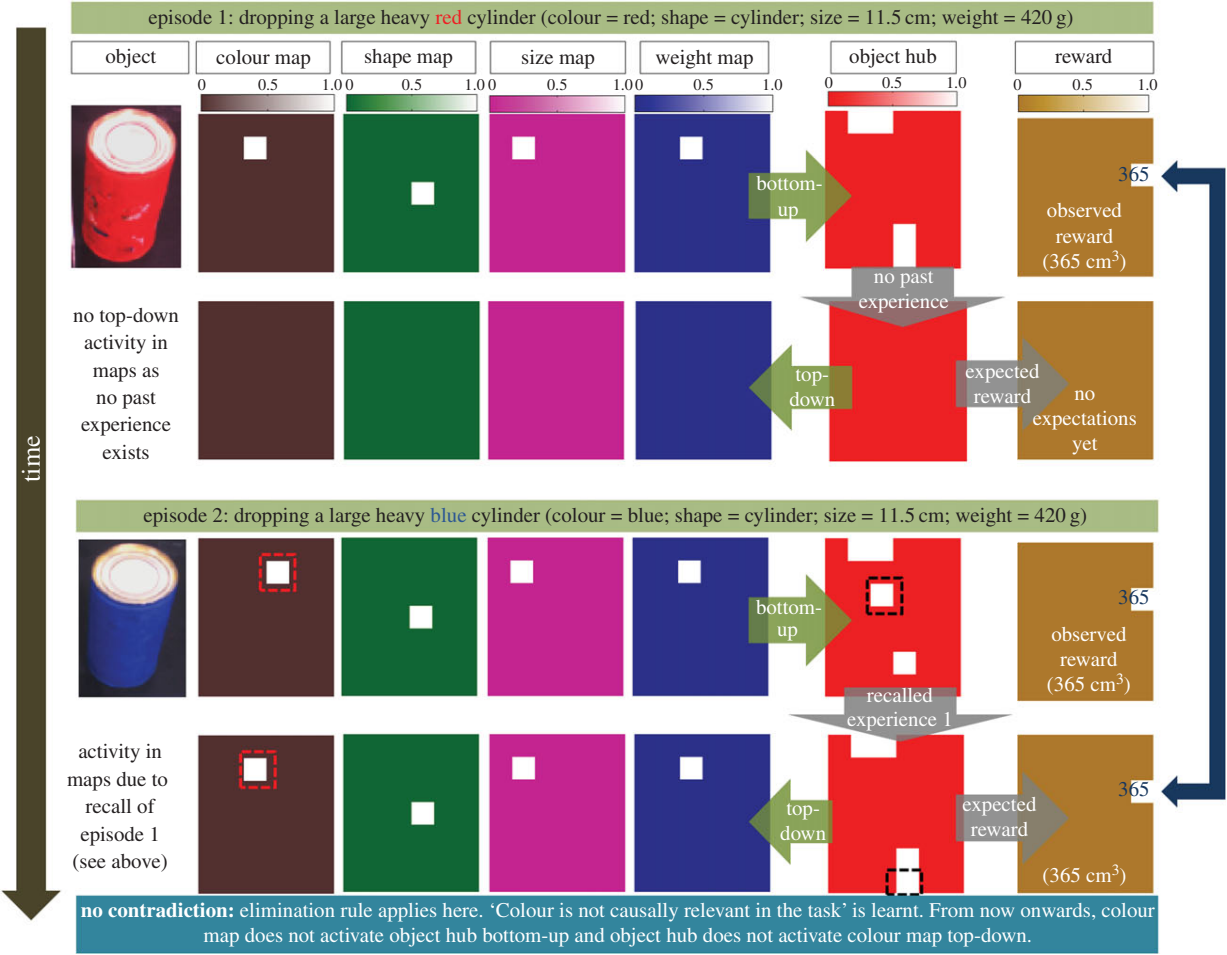
Abstracting colour is an irrelevant property in the Aesop's Fable task. The figure shows neural activity in various property-specific maps, the object hub and the reward hub for a consecutive sequence of two experiences the robot gains by interacting with two similar objects of different colour. In each episode, the first row represents the object and the bottom-up activity of all the neural maps and hubs related to sensorimotor information of the object whereas the second row depicts top-down activity in the maps and hubs due to recalled past episodes. The winning neurons in each network are shown in white (see activity scales on top of the figure). In episode 1, the bottom-up activity (1st row) does not recall any past experience; hence there is no top-down activity (2nd row). The bottom-up activity in the object hub and the reward hub during episode 1 is finally encoded into episodic memory. In episode 2, colour of the object is different from the one explored in episode 1. Upon sensing the new object, the system recalls the experience of episode 1, infers the reward based on it and acts. No contradiction in expected and observed results is found even though there was change in colour property. Elimination rule applies to colour property; the robot learns that the colour of objects is irrelevant to the task. From now on, the object hub and the colour map will not activate each other during any bottom-up or top-down information flow.

In the first scenario (figure 5, figure 1*a*), iCub is issued the goal to reach the green ball. The motion planning system of iCub provides the information that the goal is unreachable (figure 5*a*). A large heavy red cylinder is available and detected (figure 6, row 1). Bottom-up sensory streams activate property-specific maps related to (red) colour, (cylinder) shape, (11.5 cm) size and (420 g) weight properties leading to the representation of the object in the object hub. The object hub activity leads to generation of a partial cue for the recall of any related past episodes. Since there is no previous experience in the episodic memory, nothing is recalled (by the episodic memory dynamics: see Material and methods: §5.7), there is no top-down activity in the object hub (figure 6, row 2) nor any reward expected. The robot chooses to pick and drop the object into the jar of water (figure 5*b*). The object sinks in the water displacing a volume of water of about 365 cm^3^ enough to make the floating green sphere reachable (figure 5*c*). This experience is encoded into the episodic memory: an unreachable goal (body hub state), dropping (action hub activity) a large heavy red cylinder (object hub activity), a volume of water displaced 365 cm^3^ (reward) and goal realized successfully (body hub state). Note that this is a rapid one-shot encoding of experience into memory. This first experience can be recalled in the future to generate predictions or foster further learning and abstraction.

#### 3.1.2. Episode 2

The robot is presented with a blue cylinder (different colour) but of the same size and weight as in episode 1. Bottom-up perception leads to the distributed representation of this object in the object hub, which is a source of partial cue to the episodic memory system. Note that the object hub representation (figure 6, row 3) is partially similar to the object the robot had interacted with in episode 1 (figure 6, row 1). Based on the partial cue, the only past experience of the robot with the red cylinder is recalled (i.e. episode 1: figure 6, row 4). Recall of past experience is also the source of anticipating the future rewards. The robot anticipates that the large heavy blue cylinder would displace the same amount of liquid (365 cm^3^) as recalled from its past experience with the red cylinder. The anticipation turns out to be correct once the robot actually drops the object into water. Note that in this case, there was no contradiction between the expected behaviour of the object (reward hub activity in the recalled past experience) and the observed behaviour. In sum, there is a change in object property (colour) that did not cause any contradiction in the expected behaviour. Elimination rule applies here. Comparing the bottom-up real experience with the top-down anticipation due to recall of past experiences from memory (figure 6, rows 3 and 4), and applying the appropriate learning rule (elimination), the robot learns that colour is not a causally dominant property as far as the Aesop's Fable task is concerned.

### 3.2. Learning that weight is a causally dominant property

#### 3.2.1. Episode 3

**Figure 7.**
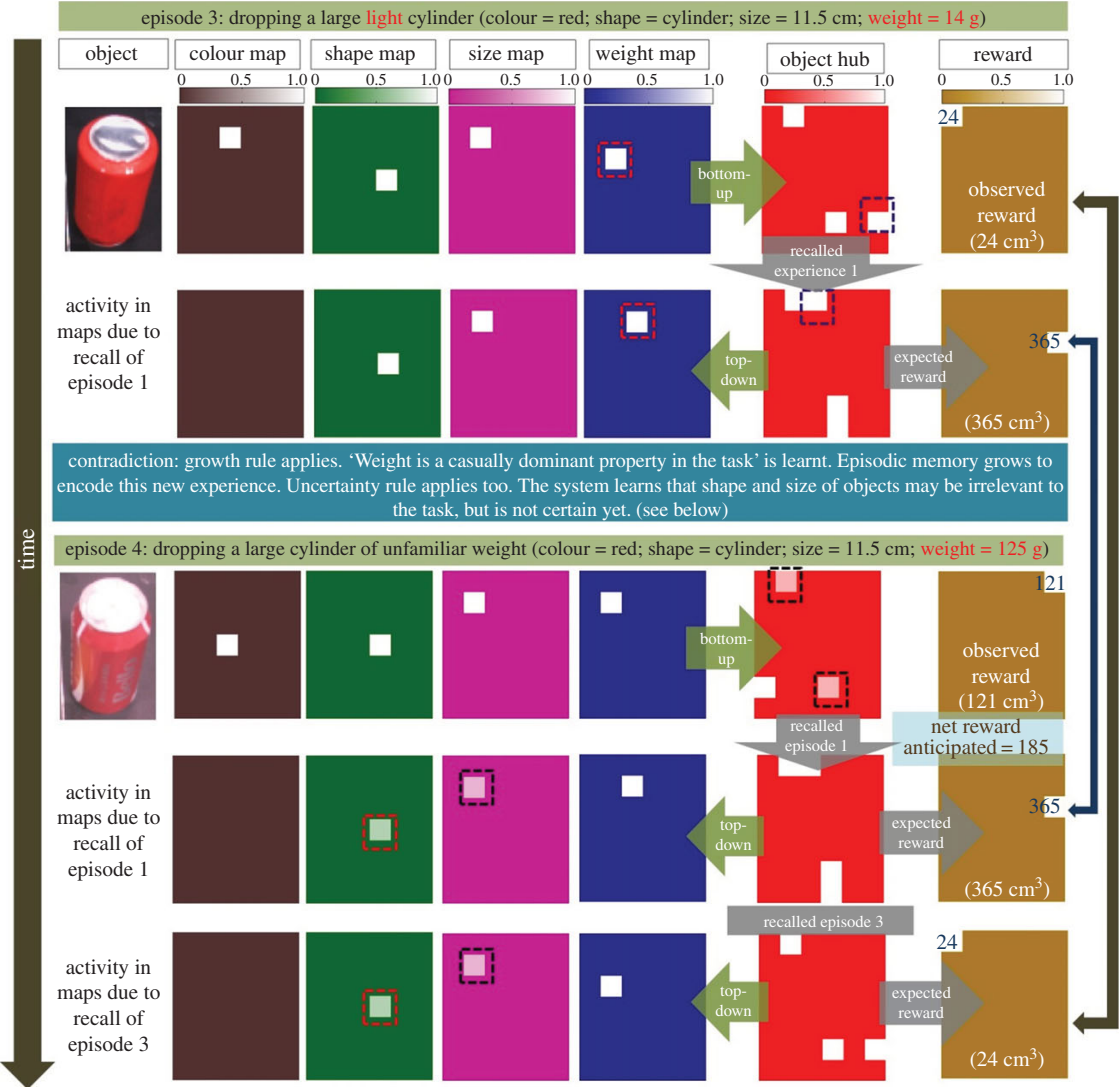
Abstracting weight is a dominant property and size is irrelevant. Similar to figure 6, this figure shows neural activity in various property-specific maps, the object hub and the reward hub for two more episodes. In episode 3, an object of a different *weight* (14 g) is presented. Bottom-up activity leads to the recall of the only episode stored in memory (episode 1) and hence the anticipated reward is that achieved in episode 1. However, upon interaction with the object, the robot observes a contradiction in anticipated and observed rewards. Comparison of top-down and bottom-up activity in different maps shows the weight property has changed. Growth rule applies; the robot learns that weight is a causally dominant property and encodes this new experience into memory. Uncertainty rule also applies. Shape map and size map showed no change; hence connectivity between the hub and these maps is marginally reduced. In episode 4, another novel object of a different weight (125 g) is presented. The corresponding bottom-up object activity recalls the encoded two experiences of the past from episodic memory. The net anticipated reward is calculated using a weighted averaging of both the rewards corresponding to the two memories. Anticipation does not turn out to be accurate. Growth rule applies. Hence this episode is also encoded into memory to allow better anticipation in future. Uncertainty rule applies too to shape and size properties.

After learning the causal irrelevance of colour, the robot is presented with a very light cylinder (14 g). Figure 7 (episode 4) shows the bottom-up and the top-down activations in different maps. Bottom-up activity leads to recall of episode 1 (as the shape and the size of the presented object still match that of the object encoded before: there is partial similarity hence generating partial cue). A high reward (volume of water displaced) of 365 cm^3^ is anticipated as before. However, the real experience with the light cylinder shows only a small amount (24 cm^3^) of water is displaced. A contradiction between the expected and the observed behaviour has occurred. The comparison of the bottom-up activity and the reconstructed top-down activity reveals a difference in weight maps. Note that the colour map is no longer activated top-down (as it is causally irrelevant). The Growth rule applies here because a change in the weight property causes contradiction between the anticipated and the observed behaviour. Applying the Growth rule, the new experience is encoded into the episodic memory which can be recalled next time for better prediction. Further, in this interesting case, activity in the shape map and the size map showed no change even though there was a contradiction between the expected and the observed behaviour. Hence the Uncertainty rule applies too: as the robot still has no experience or complete knowledge of the causal relevance of object size or shape. The system partially believes at this point that shape and size of the object may not be relevant in causing water displacement.

#### 3.2.2. Episode 4

To see the effect of the rules applied above in terms of map activity, we proceed to episode 4 where the robot is presented with another object of same shape and size as in episode 3 but of a different weight (125 g). As shown in figure 7 (episode 5, row 1), there is reduced activity of neurons in the object hub connected to shape and size maps when activated bottom-up (the effect of Uncertainty rule applied during episode 3). Both episode 1 and episode 3 are recalled because bottom-up activity due to shape and size maps (even though reduced) still matches that of objects encoded into memory previously, hence generating partial cues. Since there are two recalled memories anticipating different rewards, the system takes a property-weighted average of the two recalled rewards as its anticipation (see Material and methods: §5.8). In this case, the net anticipated reward is 185 cm^3^, however, the observed displacement in the volume of water is 121 cm^3^. The robot sees a contradiction again and by comparing the bottom-up and top-down activations in both maps, finds a change in the weight property. The Growth rule applies; the experience is encoded into episodic memory to be recalled later contextually for a better prediction.

### 3.3. Learning that size is not a causally dominant property

#### 3.3.1. Episode 5

The robot is presented with a novel object (a small heavy cylinder). This time the weight and the shape of the object are the same as that of the object in the last experience (episode 4) but the size (6.7 cm) is different to that of all the objects encountered before. Bottom-up activity due to shape and weight maps recalls experience 4 only. The robot expects the reward should be same as in episode 4, i.e. 121 cm^3^. This turns out to be a correct anticipation. The elimination rule applies again leading to the inference that size is not causally relevant.

Through rapid encoding of novel episodic events and gradual assimilation of causal relations into memory, the robot has learnt many things so far: colour of objects does not affect the amount of water they displace nor does the size of the objects; weight of the objects determines how much fluid is displaced; and heavier objects displace more water than lighter objects. The learning process continues, more episodes follow as shown in the electronic supplementary material, figure F1. Whenever the robot faces a contradiction between its expectation and real observation, the new experience is encoded. At the same time, for properties that show no change (like shape) even during contradiction, the certainty of their irrelevance keeps increasing. At the same time, the robot can anticipate the behaviour of a wide range of objects with different sizes, colours and weights.

**Figure 8.**
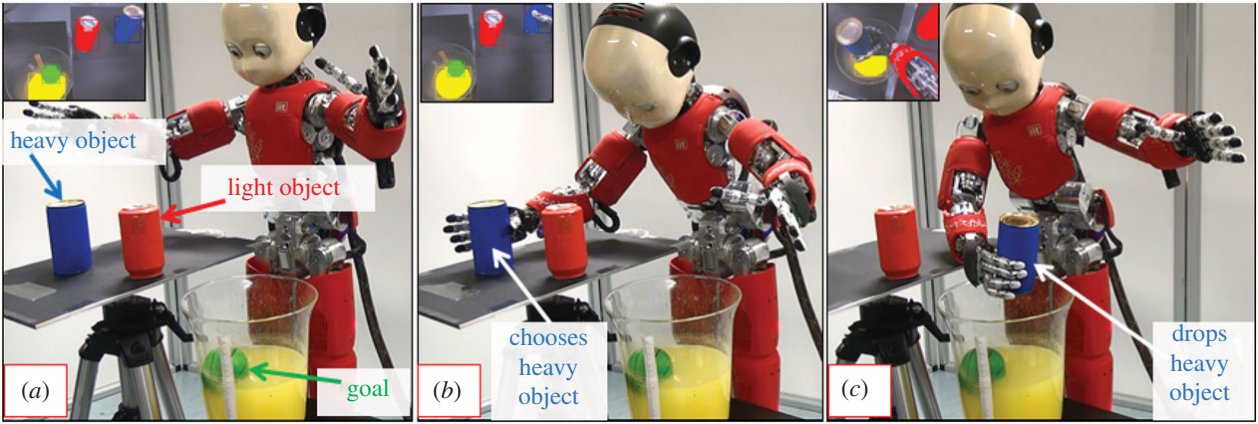
Causal inference in goal-directed tasks. (*a*) The green ball (goal) is unreachable. Two objects are present in the scene (blue and red cylinder). (*b*) Recalling past experiences, the robot infers/simulates the consequences of exploiting them and (*c*) chooses the heavier blue object with the prediction that the water displaced would make the ball reachable. The figure illustrates, how past experiences are opportunistically exploited in novel situations to modify the present environment and make it more conducive towards realization of one's internal goals: a central feature of goal-oriented reasoning. The humanoid robot used in the experiments was developed at Istituto Italiano di Tecnologia (IIT) through the EU funded project RobotCub (Material and methods: §5.1).

Interestingly, the cumulative learning of causal relations (e.g. weight of objects effects the amount of water they displace), enables the system to infer what different objects can afford in the context of a sought goal, as depicted in figure 8 where iCub infers that dropping a heavy object will make the target reachable while a light object will not, thus making intelligent choices similar to corvids [1] and children [6]. For example, in [5], the authors report that through experience over several trials of dropping objects in water, Eurasian jays learn to choose sinking objects instead of floating objects to retrieve otherwise inaccessible food rewards (analogous to the green ball that is the goal of the robot). Similarly, in [6] children are either trained or motivated to try and drop stones into water to retrieve a floating reward and hence learn the causal nature of the task over multiple trials (analogous to the way iCub learns by exploring with different objects over multiple experiences).

### 3.4. For causal learning, order of experiences does not matter

**Figure 9.**
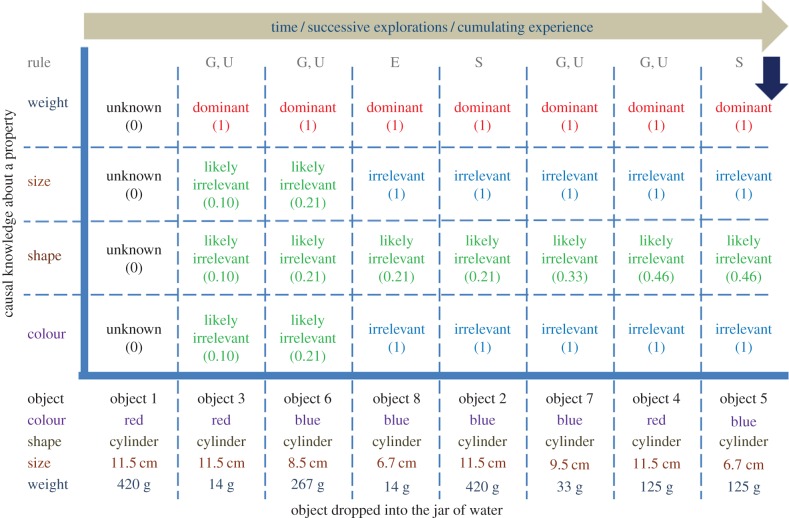
For causal learning, order of experiences does not matter. Plots the growing causal knowledge regarding four properties (colour, shape, size and weight) in the system versus the objects explored over increasing time. The plot corresponds to a different order in which objects are explored than the order discussed in the text (from episodes 1–5) and figures 6 and 7. Causal knowledge in the system regarding each property at any given time is either *Unknown* meaning nothing is known about the causal effect due to the property (depicted by 0 in the plot); or has been learnt to be *Dominant* or *Irrelevant* (depicted by 1); or the system expects the property to be *Likely Irrelevant* (by a certainty value between 0 and 1). Learning rules that apply when a new object is explored are indicated in each column using letters G, U, E, S; G stands for Growth rule, U for Uncertianty rule, E for Elimination rule and S for status quo rule. As learning progresses, weight becomes known as a dominant property, colour and size properies are eliminated and the system is still not certain about the effect of shape but the certainty that it is likely causally irrelevant keeps increasing. Thus, the system has attained same amount of causal knowledge as attained in the order discussed in the text, even though the individual episodes in which causal relevence of a property is known varied in temporal order of experience.

A feature of the proposed framework is that for the learning rules to abstract the causal knowledge, the order in which experiences are acquired does not matter. Figure 9 depicts the results of cumulative learning when objects are presented in a different order than that discussed so far through episodes 1–5 (also see electronic supplementary material, figure F1). The figure plots the amount of causal knowledge about the object properties learnt by the robot against time as new objects are explored successively. Initially when nothing is known, causal knowledge related to each property is zero. After interaction with a range of objects, a property is known to be dominant or irrelevant (represented by 1); or the robot may not be certain about the causal relevance of a property and this uncertainty is represented by a number between 0 and 1. Causal learning of different object properties occurs at different times; for example, in episodes 1–5 discussed before, causal learning of the irrelevance of colour is learnt in just the second experience, whereas in figure 9 this knowledge is abstracted in the fourth experience in order (using object 8). However, the amount of causal knowledge upon interaction with a set of objects is the same, even if the objects are explored in different orders. Plots for four (randomly generated) orders of presentation of objects showing the same end-results are provided in the electronic supplementary material, F1–F4.

### 3.5. Growing experience paves way for better prediction (towards inferring the Archimedes' principle)

**Figure 10.**
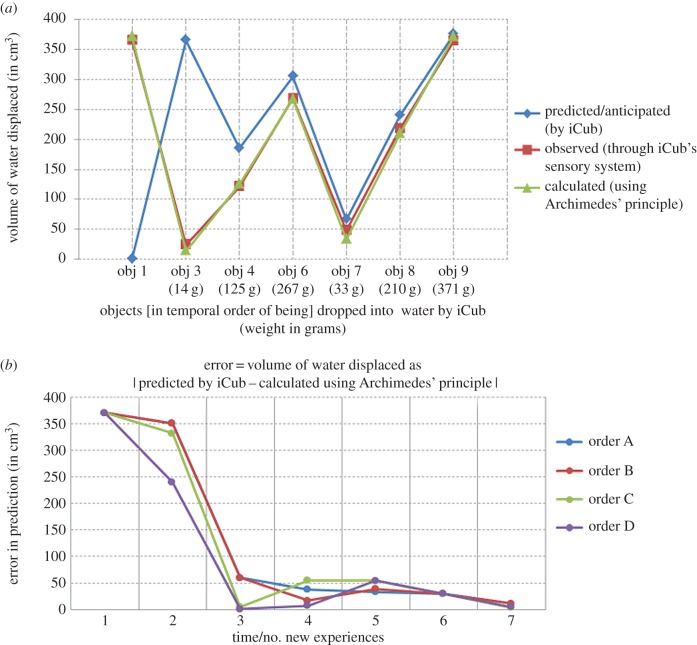
Towards inferring Archimedes' principle. (*a*) Shows a plot with three curves against the objects of different weights that are explored successively over time: a blue curve indicating the volume of water predicted by the robot that will get displaced (i.e. reward) by dropping the object into water; a red curve indicating the volume of water that was displaced as observed by the robot after it dropped the object into water; a green curve indicating the calculated volume of water displaced using Archimedes' principle. The blue curve approaches closer to the red and green curve as more and more objects are explored indicating the robot's prediction comes close to the physical law, i.e. the Archimedes principle. Note that the order of objects is the one described to explain the learning process in the text and electronic supplementary material, figure F1 and does not include the objects that have the same weight values as those explored before (for such objects prediction is accurate). (*b*) A plot of decreasing error in the predicted displacement in the volume of water by dropping an object and actual calculated displacement using Archimedes' principle. Four curves corresponding to four different orders in which the objects were presented to robot for exploration. In all sequences of exploration, the prediction approaches closer to reality as newer experiences are gained over time.

Since the computational framework implementing episodic memory enables recall of contextual past episodes, the system becomes endowed with a great power of anticipation. For novel objects of different colours and sizes (i.e. the causally irrelevant properties) the robot can easily recall the past episode and predict accurately if an object of the same weight (i.e. the causally dominant property) has been explored before. However, in cases when the system is presented an object of a weight never experienced before, all the past experiences (due to the same shape) will be recalled and a weighted averaging of the rewards expected due to these past experiences can be used as an estimate of net anticipated reward. As the number of experiences with objects of different weights increases, the accuracy in the prediction of reward increases significantly. Figure 10*a* shows a plot of the increasing accuracy in prediction of the reward (i.e. the volume of water displaced by an object when dropped into water). The plot shows three curves: the blue curve corresponds to the reward value predicted by the robot for the object to be dropped into water; the red curve corresponds to the reward observed by the robot after the object is dropped into water and the green curve corresponds to the volume of water displaced measured using the physical law, i.e. Archimedes' principle. As is obvious from the graph, over the growing number of experiences, the anticipated volume (blue curve) approaches closer and closer to the observed volume (red curve) and calculated volume (green curve), i.e. the Archimedes' principle. In figure 10*b*, we plot the error in prediction, which is the difference between the reward value predicted by the system and the calculated reward value using the Archimedes' principle, against the number of experiences as they grow in time for four different orders of presentation of objects (discussed before). The results clearly show error in prediction rapidly decreasing in all cases.

## 4. Discussion

A humanoid robot infers Archimedes' principle, so what? Firstly, an ensuing significance of this work is in the application domain particularly in the context of applied cognitive robotics. Any purposeful action of a robot situated in a physical environment is only possible if it is able to abstract and exploit the underlying causal relations between objects in the environment and its own actions. From a practical perspective, artificial agents, robots endowed with this ability have a critical cognitive edge while acting, assisting in unstructured spaces that are abound with diverse physical relations. In particular, the framework has significant utility in a range of day-to-day object-manipulation tasks such as pushing, pulling, lifting, inserting, rotating, learning to classify/use different objects as appropriate tools to realize goals, both in the case of an assistive robotic companion in a domestic environment or an industrial assembler platform for manufacturing. Consider that even today, almost all assembly tasks that have been automated are specific solutions to well-defined problems (like single-purpose systems and not general-purpose systems), with minimal learning capability from cumulative sensorimotor experiences and use of such knowledge in novel unexperienced situations. Automated industrial assembly technology still predominantly uses detailed information about objects, with detailed assembly action sequences to be executed on them to achieve the necessary reliability, accuracy of task goals. Even minute disturbances in the work-cell of a robot, introduction to novel objects or even small variations of the same task can easily lead to failures. Thus, this lack of learning and cognitive flexibility increases the cost of assembly systems and consequently of products produced by industry.

Several recent roadmaps, for example, H2020 Factories of the Future [49], are emphasizing the need to alleviate this lacuna in order to bring in greater flexibility, autonomy instead of automation in the assembly chains. The ability to swiftly and cumulatively learn by interacting, abstract underlying cause–effect relations and exploiting such knowledge in novel situations as explored in this article is a critical functional repertoire in this direction. Some preliminary work in porting/validating the framework in real-world industrial assembly is already underway [44] reporting significant advantages, for example, 15 min to switchover/learn a new assembly task with new objects compared to more than 3 h with the industrial benchmark [50,51]. Finally, the acquired knowledge of cause–effect relations can also be exploited to infer potential consequences of others' actions on objects (for example, an interacting partner: human or another robot) in a joint goal situation (for example, actor 1 picks up a fuse, the goal is to insert into an appropriate fuse holder to assemble a fuse box). Studies from embodied cognition support the perspective that understanding others' actions may be conceived as an internal simulation that entails the re-use of our own ability to act in order to functionally attribute meaning to the actions of ‘others’, and on the way, recycling some of the same cortical/computational substrates the enable us to act ourselves [52]. While the framework has recently been ported to an industrial setting for tasks like object assemblies [44], the future extensions to the work will include robotic applications to a broader range of industrial and domestic tasks. At the same time, effective human–robot cooperation is of emerging interest in several applied domains, offering a strong potential scope for further extension of the research explored in this article.

While the applied perspective is appealing, how animals are able to learn and exploit object affordances and causal structure in their environments through cumulative learning experiences is a fundamental question in cognition. In this context, our work takes the topic of affordances from the level of object–action to the level of property–action, in line with emerging studies from neurosciences, mainly, the distributed property-specific organization of sensorimotor knowledge in the neo cortex and the brain's DMN, which emphasizes a shared cortical basis for recalling the past and simulating the future. Further, the implicit advantage is that the learnt property–action relations can be effortlessly generalized to a domain of objects for which a cognitive agent need not have any past experience/learning but, nevertheless, share the property. We must mention that this is not a new concept in psychology. Rather, it is best reflected in the writings of psychologist William James [53] where he illustrated the different uses of a book (a thing that gives knowledge, a paper weight, an instrument to swat a mosquito) based on one's immediate goals. Our work in this context both brings together emerging trends in neurosciences as well as concepts from psychology to provide a neutrally plausible computational basis for causal learning and inference mechanisms in both natural and artificial agents. Work is ongoing to mirror advanced experiments on the Aesop's Fable such as the U-tube task and provide a computational basis for differences observed in corvids and children in these experiments [1,6]. At the same time, we believe further research in this direction has the potential to provide a bioinspired and embodied framework for reasoning by analogy (attributing causality to a novel class of objects based on what has been learnt and experienced in the past).

A central feature of the proposed architecture is that it shares the same neural substrates (property-specific maps and hubs) during both top-down and bottom-up information flows. A bulk of studies surrounding embodied cognition provides direct evidence for this [52,54,55]. However, it is not clear why the neural substrates should be shared and what are the computational advantages. Our work clearly demonstrates that such kind of sharing simplifies the comparison between what has been experienced in the past (i.e. reconstructed through memory) with what an embodied agent is presently experiencing, since both top-down and bottom-up information are brought down to a common platform (i.e. the shared neural substrate: property-specific maps and hubs), thereby triggering mechanisms for abstraction and inference. In this context, this is a computational emulation of cognitive dissonance [56], but at the same time taking into account more recent accounts of the underlying neuroanatomical basis and cortical interactions that give rise to such mechanisms [36,48,57]. Further emerging studies from neurosciences point to a homologous cortical network in rats, corvids and humans underlying such functions (see, [58] for a review). In this context, this article provides a similar brain-guided framework to enable cognitive robots to exhibit similar behaviours while learning cumulatively and realizing their goals. The property-specific organization of conceptual information and the learning rules applied herein are domain agnostic, independent of the task at hand. The same learning rules and the property-specific conceptual organization have been employed in a simpler model to test learning of property–action relations in a task of pushing [59] where the robot cumulatively learns and abstracts causally dominant properties that influence the motion of various objects when forces are exerted on them. For example, the robot learns that large cylinders move faster than large cubes when pushed. Small cylinders move even faster than large ones. Interestingly, in an embodied framework this brings in the power to make transitive inferences (such as small cubes should move faster than larger ones) without any need for symbolic processing.

Finally, the work presented emphasizes that reasoning and learning always have to go hand in hand and grow cumulatively and continuously during the lifetime of a learner, be it any natural or artificial cognitive agent. While more experience drives better reasoning and inconsistencies in reasoning drive new learning. This interesting aspect of the cumulative development and living in uncertainty is highlighted and explored in this article, through the playful Aesop's Fable scenario, where at every moment the robot is acting with partial knowledge because not everything is known and not everything has been experienced, and culminates in approximately inferring the Archimedes principle!

## 5. Material and methods

### 5.1. Icub, the Humanoid platform

The iCub is a small humanoid robot of the dimensions of a 3.6-year-old child and designed by the RobotCub consortium (http://www.robotcub.org), a joint collaborative effort of 11 research groups in Europe, Japan and the USA. iCub was developed to serve as a test-bed for research into embodied cognition. The robot is open-source, with the hardware design, software and documentation all released under the GPL license. A copy of the license can be found at http://www.robotcub.org/icub/license/gpl.txt. The robot has 53 actuated degrees of freedom: seven in each arm, nine in each hand, six in the head (three for the neck and three for the cameras), three in the torso/waist and six in each leg.

### 5.2. Sensory processing

#### 5.2.1. Colour

The colour of objects is analysed by a colour segmentation module based on Markov random fields [60] which returns a triad of RGB values. These triad forms the input to the colour map.

#### 5.2.2. Shape

At the level of the concept system, information related to object shape is passed as 120-bit vector unique for each shape (like an abstract identifier of the object). In this way, the complexity of shape analysis is abstracted from the concept system.

#### 5.2.3. Size

Size-related information is organized into in a map coding for magnitude (the maximum length of the object across any axes in Cartesian space: e.g. S1) and proportion (i.e. the ratio of the maximum length with respect to lengths in the other two axes, e.g. S2). S3 relates to orientation that is not a property of the object itself but rather is relative to the frame of reference of the observer. This kind of organization of size-related information is partly inspired by recent evidence related to representation of magnitude in the parietal cortex [61]. There are several advantages of this scheme in terms of inferring what can be done with different objects that may be indistinguishable through colour or shape (e.g. consider a green cuboid and a green stick: both have same shape and colour; what distinguishes them is the abstract magnitude and proportion: the former can be used to build a stack the latter as a tool to pull an unreachable reward). However, in the context of this paper, since only cylinders of varying lengths (but same radius) were used, only the map coding for magnitude was employed in the framework.

#### 5.2.4. Word

Word information is the input directly via the teacher typing on the keyboard and converted into vectors on the basis of letter usage frequencies in the English language [47]. From an application perspective, this incorporation of little linguistics (that is grounded in sensorimotor experience of the learner) endows the architecture with a measure of user friendliness allowing easy interaction.

#### 5.2.5. Weight

Weight of the objects is estimated from measures of torques in force/torque sensors of iCub specifically those for the right arm.

### 5.3. Associating names/words with object properties

Associations between different perceptual properties such as object colours (or shapes) and their names (given by the user) are learnt through temporal coincidence. That is, if neurons in different self-organizing maps are concurrently active (within a temporal window), then they become associated to each other through object hub using the dual-dyad connectivity (red arrows in figure 1). Practically, this is accomplished by presenting the robot with an object having the property that is to be learnt (say green colour). The object property (e.g. colour) is analysed bottom-up by the sensory layer resulting in an output vector that is fed to the corresponding SOM (e.g. colour map). In the same temporal window of integration, the teacher inputs the name or word (e.g. word ‘green’) for the corresponding property, which serves as input to the word SOM. As a result, there is some neuronal activity in each of the two maps. The net activity in the property-specific maps and the word map forms the bottom-up input to the object hub. The dual-dyad connectivity matrices between the maps and the object hub are randomly initialized at the start. The learning rule to connect SOMs with the object hub is: if the net activity due to a neuron *i* and a neuron *j* winning in the colour and word SOMs, respectively, manages to activate a neuron *k* in the object hub, make W*_ik_* = 1 and W*_jk_* = 1.

### 5.4. Self-organizing map learning procedure

The SOM learning process uses standard methods as discussed in previous work [62,63]. In short, it can be accomplished in two steps:

1. Find the neuron *i* that shows maximum activity for the incoming sensory stimulus *S_t_* at time *t*. This also implies that neuron *i*'s sensory weights *s_i_* such that 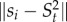 have the smallest value, among all neurons existing in the respective SOM at that instance of time.
2. Adapt the sensory weights of the winning neuron in a Hebbian fashion by bringing the sensory weights *s_i_* of the winner *i* closer to the stimulus *S_t_*. This simply has the effect that in future instances, the neuron *i* actively codes for the particular sensory stimulus *S_t_*. In this way, neurons in different property-specific maps that have sensory weights closest to the incoming input sensory vector start representing these signals.

### 5.5 Network dynamics of object hub–property-specific maps

Let *N* be the number of neurons in a SOM or a hub. Let *h_i_* be the activity of the *i*th neuron in the provincial hub and *x*_prop_ be the activity of a neuron in any of the property-specific SOMs connected to the object hub (in this case word, colour, shape, size and weight SOMs). Let *W*_prop,hub_ encode the connections between the property-specific maps and the object hub. *W*_prop,hub_ is a *N*_OjectHub_ × (*N*_colour_ + *N*_shape_ + *N*_size_ + *N*_weight_ + *N*_word_) matrix, learnt as explained in the main text (see the Results section). Its transpose encodes backward connectivity from the hub to individual maps. The network dynamics of hub neurons is governed by:5.1
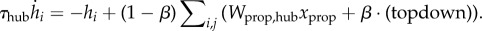


Topdown indicates activity due to recalled memory from the past. *β* = 0 means that the system operates only on real sensory input, whereas *β* = 1 indicates the system is modulated top-down only. The dynamics of neurons in the property-specific maps is given by:5.2
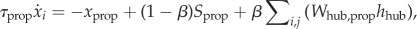
where
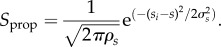
A detailed description of this process and implementation can be found in [45].

### 5.6. Encoding new experiences in episodic memory

New experiences gained by the robot are learnt by updating connections between the neurons in the episodic memory network in the following way. Let *V*_new_ be a one-dimensional vector representing the activity of *N* (*N* = 1000, here) neurons shown as a 20 × 50 matrix. Let *T* denote the connectivity matrix between the *N* neurons that represents the strength of the connection between any neuron *i* to any neuron *j*. Since there are 1000 neurons, the dimensionality of *T* is 1000 × 1000. *T* is a null matrix to start with as nothing is known. Consider that a new episode represented by activity *V*_new_ has to be stored in the memory network. This is done by updating all the connections *T_i_*_,*j*_ between the *N* neurons, using a simple Hebbian rule summarized below:

If *V_i_* = 1 and *V_j_* = 1, then make *T_i,j_* = 1 (regardless of what its value was before);
Else, make no change to *T_i,j_*.

A detailed description can be found in [48].

### 5.7. Remembering past experiences from partial cues

The network dynamics to recall past experiences from partial cues is given in equation (5.3). *V_k_* is the activity in the *K*th neuron (a subset of neurons in the 50 × 20 neural episodic memory layer will be active based on the partial cue or initial condition). *T* is the connectivity matrix between the neurons learnt when the episodes of experiences are encoded in the network.5.3
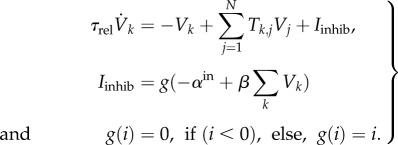
*I*_inhib_ is the current coming from the inhibition network that is modelled as a single neuron. The function of the inhibitory network is to keep the excitatory system from running away, to limit the firing rate of the excitatory neurons. At low levels of excitation, the inhibitory term generally vanishes. For all experiments, *α*^in^ was chosen as 30, *τ*_rel_ as 1000 and *β* as 3.5. For further details, refer to [48].

### 5.8. Computation of anticipated reward in case of multiple recalled memories

Let *n* be the number of top-down object hub activities reconstructed that cause top-down activity in maps. For each reconstruction *i*, we define:



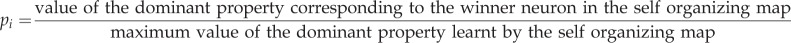

and *r*_*i*_ = reward value corresponding to object hub activity.

Then for a bottom-up object hub activity with the corresponding property fraction *p_x_*, the anticipated reward is given by:
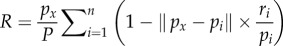
where
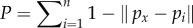


## Supplementary Material

Supplementary figures, Open Source sofware

## Data accessibility

A short video clip of the experiments with the humanoid is provided in the electronic supplementary material. A complete set of videos documenting the cumulative learning, with audio from the software listing different critical events (as described in flowchart in figure 4) is available in a YouTube channel, Re-enacting the Aesop's Fable on iCub humanoid: https://www.youtube.com/playlist?list=PLIfoHEM1gr24EniCzBuUxZ2tqNpQA8QQm. An open-source C++ software implementation of the proposed framework is available from the iCub SVN repository accessible at https://svn.code.sf.net/p/robotcub/code/trunk/iCub/contrib/src/morphoGen/.
