# Supplementary material for: Humanoid infers Archimedes' principle: understanding physical relations and object affordances through cumulative learning experiences
Source: J R Soc Interface. 2016 Jul;13(120):20160310. doi: 10.1098/rsif.2016.0310 (PMC4971221; doi:10.1098/rsif.2016.0310)
Supplement: Supplementary figures, Open Source sofware [file rsif20160310supp1.pdf]

# Supplementary Information

## Humanoid infers Archimedes’ principle: From cumulative exploration to abstraction of underlying causal relations and affordances

*Ajaz Ahmad Bhat, Vishwanathan Mohan, Giulio Sandini, Pietro Morasso*

*Robotics, Brain and Cognitive Science department, Istituto Italiano di Tecnologia, Italy*

**This supplementary document includes a) additional figures demonstrating that order of experiences does not matter for causal learning; b) a legend for the supplementary video documenting the learning process; and c) link to the software code provided open source for reuse and distribution.**

## 1. Supplementary Figures F1, F2 and F3

**For causal learning, order of experiences does not matter.** Plots showing the growing causal knowledge regarding four properties (color, shape, size and weight) in the system versus the objects explored over increasing time. The plot corresponds to three different orders in which objects are explored other than the order discussed in the main text. See **Figure 9** and relevant text in the article for more details. The last column of these plots depicts clearly that whichever order is chosen, the net causal knowledge attained is the same.

### 1.1 Supplementary Figure F1

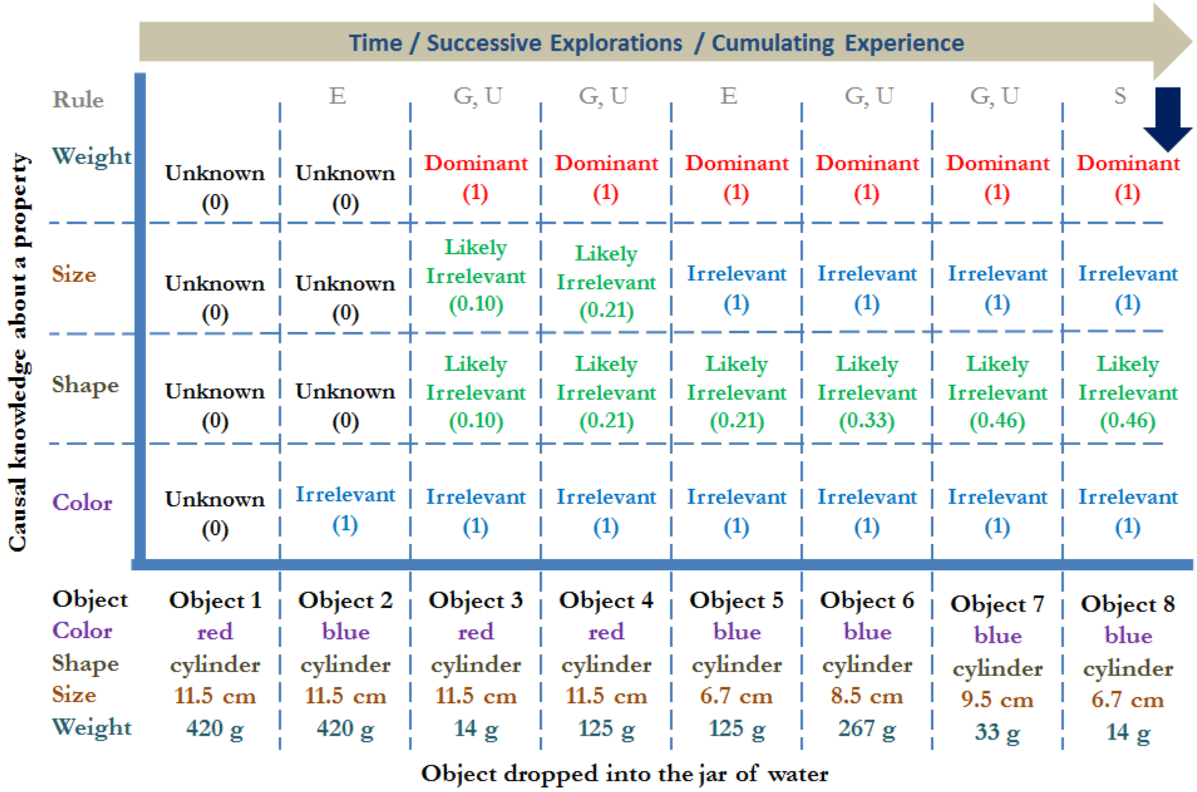

## 1.2 Supplementary Figure F3

Time / Successive Explorations / Cumulating Experience

Causal knowledge about a property

| | | | | | | | | |
|---|---|---|---|---|---|---|---|---|
| Rule | | G, U | G, U | G, U | E | S | S | G, U |
| Weight | Unknown (0) | Dominant (1) | Dominant (1) | Dominant (1) | Dominant (1) | Dominant (1) | Dominant (1) | Dominant (1) |
| Size | Unknown (0) | Likely Irrelevant (0.10) | Likely Irrelevant (0.21) | Likely Irrelevant (0.33) | Irrelevant (1) | Irrelevant (1) | Irrelevant (1) | Irrelevant (1) |
| Shape | Unknown (0) | Likely Irrelevant (0.10) | Likely Irrelevant (0.21) | Likely Irrelevant (0.33) | Likely Irrelevant (0.33) | Likely Irrelevant (0.33) | Likely Irrelevant (0.33) | Likely Irrelevant (0.46) |
| Color | Unknown (0) | Likely Irrelevant (0.10) | Likely Irrelevant (0.21) | Likely Irrelevant (0.33) | Irrelevant (1) | Irrelevant (1) | Irrelevant (1) | Irrelevant (1) |
| Object | Object 2 | Object 7 | Object 8 | Object 5 | Object 3 | Object 1 | Object 4 | Object 6 |
| Color | blue | blue | blue | blue | red | red | red | blue |
| Shape | cylinder | cylinder | cylinder | cylinder | cylinder | cylinder | cylinder | cylinder |
| Size | 11.5 cm | 9.5 cm | 6.7 cm | 6.7 cm | 11.5 cm | 11.5 cm | 11.5 cm | 8.5 cm |
| Weight | 420 g | 33 g | 14 g | 125 g | 14 g | 420 g | 125 g | 267 g |

Object dropped into the jar of water

## 1.3 Supplementary Figure F4

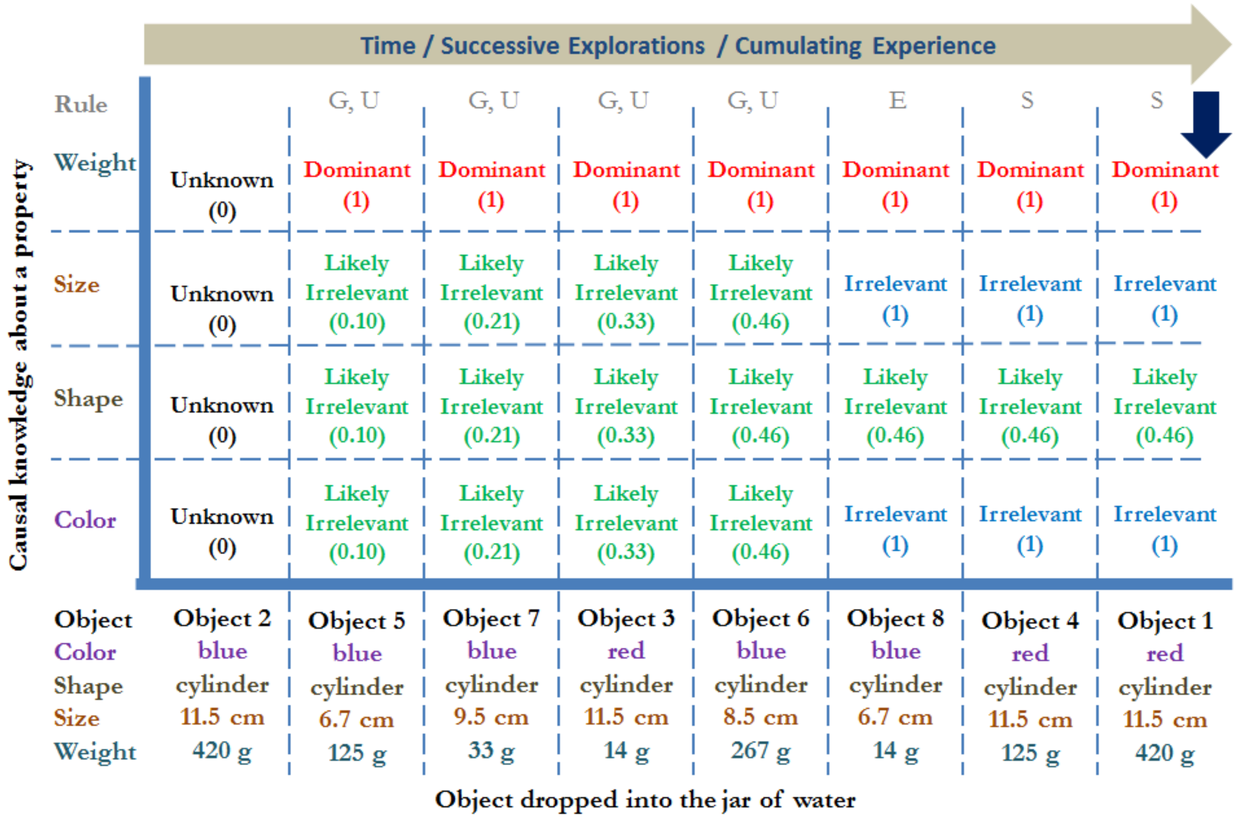

## 2. Supplementary Video V1 Legend

This short video shows some clips of the experiments carried out on a humanoid robot, iCub. The video documents some key stages of cumulative learning process while the robot learns the causal relevance of different object-properties and gradually grows its causal knowledge towards inferring the Archimedes' Principle. A complete set of videos documenting all the experiments can be found in a YouTube playlist at https://www.youtube.com/playlist?list=PLIfoHEM1gr24EniCzBuUxZ2tqNpQA8QQm. This video on iCub humanoid robot is released under the following license compatible with GPL:

**Copyright** (C) <2016> <Istituto Italiano di Tecnologia>
**Author**: <Ajaz A Bhat, Vishwanathan Mohan>
**Email**: ajaz.bhat@iit.it, vishwanathan.mohan@iit.it

Permission is granted to copy, distribute, and/or modify this file under the terms of the GNU General Public License, version 2 or any later version published by the Free Software Foundation.

A copy of the license can be found at
http://www.robotcub.org/icub/license/gpl.txt

This program is distributed in the hope that it will be useful, but WITHOUT ANY WARRANTY; without even the implied warranty of MERCHANTABILITY or FITNESS FOR A PARTICULAR PURPOSE. See the GNU General Public License for more details

To view a copy of GPL license visit http://www.gnu.org/licenses/gpl-3.0.en.html.

## 3. Open Source Release of Software code

The C++ source code used for these experiments is also provided open source under GPL license. A copy of the GPL compatible license for reuse, distribution and modification of the software source code can be found at https://svn.code.sf.net/p/robotcub/code/trunk/iCub/contrib/src/morphoGen/license.txt.
